# Phytochemistry and Biologic Activities of *Caulerpa Peltata* Native to Oman Sea 

**Published:** 2014

**Authors:** Nasrin Movahhedin, Jaleh Barar, Fatemeh Fathi Azad, Abolfazl Barzegari, Hossein Nazemiyeh

**Affiliations:** a *Research Center for Pharmaceutical Nanotechnology, Faculty of Pharmacy, Tabriz University of Medical Sciences, Tabriz, Iran.*; b *Students’ Research Committee, Tabriz University of Medical Sciences, Tabriz, Iran.*; c *Faculty of Pharmacy, Tabriz University of Medical Sciences, Tabriz, Iran.*

**Keywords:** Caulerpa peltata, Caulerpin, MTT, DPPH, BSLA

## Abstract

General toxicity, antiproliferative, antibacterial and antioxidant activities of *Caulerpa peltata *J.V.Lamouroux (Caulerpaceae) collected from Oman Sea were investigated. Dried, ground alga was Soxhlet-extracted with hexane, dichloromethane and methanol successively.

The methanol extract was subjected to vacuum liquid chromatography (VLC) fractionation on silica gel using a step gradient of different mixture of solvents. A known alkaloid, caulerpin, was subsequently isolated from the fraction eluted by ethyl acatete100%. The antioxidant activity of all extracts was assessed by using the (DPPH) assay. *Antiproliferative* activity of the *all extracts* and caulerpin against the cancerous cell line was evaluated using *MTT assay*.* General toxicity of extracts was determined using* Brine Shrimp Lethality Assay (BSLA).

*Based on our results, a weak activity* observed for all extracts in MTT assay, while they were toxic toward brine shrimp nauplii comparing to the podophylotoxin. This is the first report on phytochemistry and bioactivity of *C. peltata* which collected from Oman Sea.

## Introduction

Marine natural products have attracted the attention of the scientist due to their precious and hopeful compounds related to the fighting against cancers and other disease. During the last decades, numerous bioactive compounds have been isolated from the marine resources, have shown to possess significant pharmacological effects comparing to the current medicines (1-9) which offering a great opportunity to develop new classes of anticancer agents. It is believed that seaweeds and other marine plants, as the primary producers in the sea environment (10) provides food and many of the active nutraceuticals(11-14). Approximately 6000 species of seaweeds have been identified and categorized into three broad groups *i.e*. green (Chlorophytes), brown (Pheophytes) and red (Rhodophytes) algae (15).

The genus *Caulerpa is* nearly comprised of 60 species which are broadly distributed in tropical and subtropical waters(16). Some species of *Caulerpa* (Caulerpaceae) are edible and used as salad, however it has been shown that consummation of Caulerpa species specially during the rainy season could cause poisoning which ascribes to caulerpicin (17-18). Moreover, it has been mentioned that herbivorous fish and invertebrates avoid consuming of the Caulerpaceae and Codiaceae plants (16)*. *Algae of Caulerpaceae family produce several secondary metabolites including sesquiterpenoids and deterpenoids (16) which protects plants from herbivores. It seems that caulerpenyne, a sesquiterpen, plays a major role in chemical defense (19). In addition triterpenes; squalene, squalene epoxides, sterols, di-indolo pigments; caulerpin and its analogues, caulersin (20), a mixture of ceramides derivatives; caulerpecin (21) are the other secondary metabolites that isolated from different *Caulerpa *species. Earlier studies had shown anti HSV-1 (22), antibacterial activity (23), antitumor activity (24) and plant regulatory effects (25) for caulerpin.

The previous published papers show that more than 150 species of algae grow alongside the southern shore borders of Iran (26, 27). Certainly, this situation provides a large field for studies related to the biology of algae and their phytochemistry, as well. To the best of our knowledge, there are ambient studies regarding the phytochemistry and biological activities of Oman Sea and Persian Gulf native algae. Hence, in the present work we like to report the results of a study on the chemical constituents and biological activity of an endemic green alga, *Caulerpa peltata*, collected from Oman Sea.

## Experimental

*General experimental procedures*

^1^H, ^13^C NMR and 2DNMR spectra were recorded in CH_3_OH-d_4_ on a Brucker Avance DRX 500 spectrometer operating at 500.13MHz for ^1^H NMR and 125.03 MHz for ^13^C NMR. Tetramethylsilane (TMS) was used as internal standard. Electrospray ion mass spectrometry (ESI-MS) was performed with a Bruker APEX II mass spectrometer. HPLC analysis was performed using a Dionex P580 system coupled to a photodiode array detector (UVD340S). Detection was done at 235, 254, 280, and 340 nm. Purification was performed using a LaChrom L-7100 (Merck/Hitachi) semi-prep HPLC system. A Eurospher-100 C18 column (250 × 21.4 mm, 10μ; Knauer) with a Eurospher-100 C18 (Knauer) pre-column was used. UV data for individual compounds were extracted from the online UV spectra provided by the instrument software. 

*Plant materials*

Green alga *Caulerpa peltata *J.V.Lamouroux (Caulerpaceae) was collected in November 2008 from Chabahar coast (Oman Sea, Iran) and dried at room temperature. Collected samples were determined by Mr. BM Gharanjik and a voucher specimen has been deposited in the Herbarium of Offshore Fisheries Research Center, Chabahar – Iran.

*Extraction and purification*

Dried and ground samples (100 g) were Soxhlet-extracted, with *n*-hexane, dichloromethane (DCM) and methanol (1.1 l, 8 h; each), respectively. Solvents were removed *in vacuo *by rotary evaporator at a maximum temperature of 45 °C . Each crude extract used for bioassay tests. 

The methanol extract (2 g) was subjected to vacuum liquid chromatography (VLC) on silica gel using a step gradient of different solvents mixtures (*n*-hexane: Ethyl acetate, methanol: dichloromethane and methanol: acetone). The fraction eluted by ethyl acetate 100% was subjected to semi prep-HPLC analysis, eluting with a linear gradient of methanol in nano-pure water (elution program: 0-5 min, 10% methanol in water; 5-35 min, 10-100% methanol in water; 35-45 min, 100% methanol in water; flow rate: 5 mL/min; detection at 235 and 280 nm) afforded 20 mg of a toxic red pigment (Rt is 29.49 min) as pure compound. The spectral data of this pigment was as: orange red prisms,”online UV λ _max_ (nm): 220,270,290 and 317; EI-MS m/z: 398.1 [M^+^] (100), 366 (17.3), 338(12.6), 306 (31), 279 (59), 251 (10) and 139 (20); ^1^H and ^13^C -NMR data, see Table 2. 

*Brine shrimp Lethality assay*

General toxicity of the extracts was assessed on brine shrimp nauplii according to Meyer *et al.* (1982) with modifications (28). Artificial sea water was prepared by dissolving ca.38 g sea salt per liter of water. Brine shrimp eggs (*Artemia salina*) were hatched in a conical flask containing artificial sea water during 48 h incubation under a bright light in a water bath (29 ^o^C). Dimethyl sulfoxide (DMSO) was used to dissolve the extracts and sufficient artificial sea water was added to obtain a concentration of 5 mg/mL (stock solutions). Serial dilutions were prepared (1000 μg/mL, 100 μg/mL, 10 μg/mL) from the stock solutions. About 10–15 nauplii were added with the aid of a Pasteur pipette to each set of tubes containing the diluted extracts. Controls containing 1000 μL of DMSO in seawater were included in each experiment. Podophylotoxin dissolved in DMSO (1 mg/mL) was used as a positive control. Twenty-four hours later, the number of survivors was counted (each experiment was done in triplicate) the median lethal concentration (LC_50_) were calculated by Probit Analysis (Finney, 1971).

*MTT assay*

Cytotoxicity of the *extracts* against the cancerous cell lines compared with normal cells was assessed using *MTT assay* according to the method described by Carmichael (1987). Cell viability was determined by spectrophotometric determination of accumulated formazan derivative in treated cells at 560 nm in comparison to control cell (29). L5178Y mouse lymphoma cells were grown in Eagle’s minimal essential medium supplement with 10% horse serum in roller tube culture. The medium contained 100 IU/mL Penicillin and 100 IU/mL Streptomycin. The cells were incubated under a humidified atmosphere and 5% CO_2_ at 37 °C. A stock solutions of test samples to be analyzed were prepared in EtOH 96% (v/v). Exponentially growing cells were harvested and plated at 3750 cells cm^-2^ into 96-well plates. Subsequently the test samples solution was added to each well and plates were incubated at 37 °C for 72 h. A solution of MTT was prepared at 5 µg/mL in phosphate buffered saline (PBS; 1.5 mM KH_2_PO_4_, 6.5 mM, 43 Na_2_HPO_4_, 137 mM NaCl, 2.7 mM KCl; pH 7.4) and 20 µL of solution was transferred into each well. The plates were incubated at 37 °C for nearly 4 h. At the end of the incubation time, the medium was centrifuged (15 min at 210 x g) and removed. Then 200 µL DMSO was added to each well to liberate the formazan product. After thorough mixing, the absorbance was measured at 520 nm. The color intensity could be correlated with the number of healthy living cells and cell survival was calculated using the formula:

Survival (%) = [(AU−AC)/(AT−AC)]x 100 

Where in: AU is absorbance of untreated cells, AT is absorbance of treated cells and AC is absorbance of culture medium. 

All experiments were carried out in triplicate and repeated three times. As negative controls, media with 0.1% (v/v) EtOH were included in all experiment. As a positive control, kahalalide, a known cytotoxic compound isolated from *Elysia grandifolia *(29) was used.

*DPPH assay*

The ability of extracts to function as free radical scavengers was assessed by using the 2, 2-diphenyl-1-picrylhydrazyl (DPPH) assay(30). A solution of DPPH (Fluka Chemie AG, Bucks; 8 mg /100 mL) in methanol or chloroform was used. Dried methanolic extract was dissolved in MeOH (DCM and Hexane extracts were dissolved in chloroform) to obtain a concentration of 0.5 mg/mL. Dilutions were made to obtain concentrations of 5 ×10^-2^, 5 ×10^-3^, 5 ×10^-4^, 5 ×10^-5^, 5 ×10^-6^, 5 ×10^- 7^, 5 ×10^-8^ mg/mL. Diluted solutions were mixed with DPPH and after 30 min, the absorbance was recorded at 517 nm against a blank. The experiments were performed in triplicate and the average absorption was noted for each concentration. The same process was pursued for the methanolic solutions of quercetin as positive control .The free radical scavenging activity was then calculated from the difference in absorption between the test sample and the DPPH blank as follows:

 αA (%)=[(AB−AP)/(AB−APos)] x 100 

Where αA is % antioxidant activity in comparison with the positive control, AB is the absorption of the DPPH solution as blank, AP is the absorption of the test sample, and APos is the absorption of the positive control. The IC_50_ values were calculated from the curve equation.

*Antibacterial activity*

*Bacterial strains*

*Staphylococcus aureus* (PTCC1337), *Enterococcus faecium* (native isolate), *Escherichia coli* (O157H7) and *Salmonella paratyphi* (PTCC 1609) were used to assess the antibacterial effect of all extracts. Ciprofloxacin was used as positive control. 

*Minimum inhibitory concentration determination*

The antibacterial activity of hexane, DCM and methanol extracts was determined by the rapid microtitre-plate-based serial dilution method using resazurin as the growth indicator (31-32), with minor modification. The same method was applied for the pure compound. 

## Results

*Extraction and purification*

**Figure 1:**
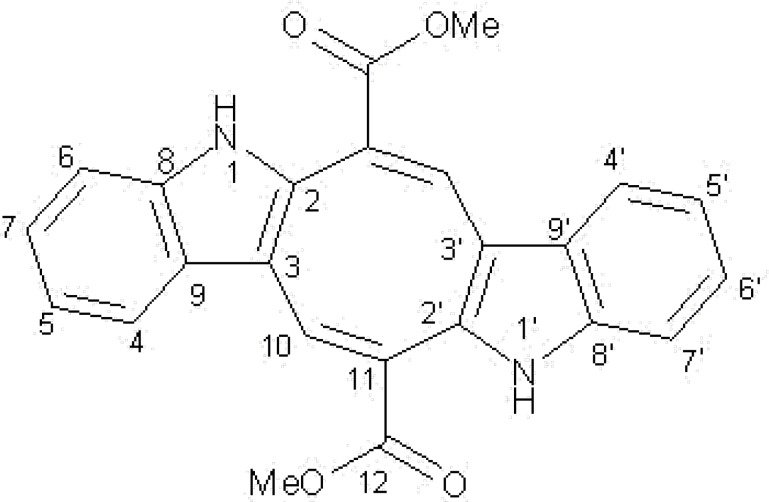
Caulerpin.

Extraction of the dried and ground *C. peltata *fronds (100 g) with hexane, DCM and methanol solvents yielded 1.5 g, 2.3 g and 16.8 g of dried extracts, respectively. The BSLA was used to assess the general toxicity of hexane, DCM and methanol extracts against the newly hatched nauplii of brine shrimp eggs (Table 1). It is apparent that the methanol extract was more toxic than the other extracts, where its activity was not comparable with podophyllotoxin effect. This finding candidate the methanol extract for more investigation. A combination of Vacuum Liquid Chromatography fractionation (VLC) on silica gel, and prep-HPLC of the methanol extract afforded 20 mg of an orange red pigment. The ESI- MS spectrum showed a [M^+^] ion at m/z 398, corresponding to a molecular formula of C_24_H_18_N_2_O_4_. Comparison of the molecular weight of pigment with the ^1^H and ^13^C NMR data revealed a symmetric structure for the compound. In the ^1^H NMR spectrum, presence of a doublet pair at 7.4 (2H, d, 8Hz) and 7.3 (2H, d, 8Hz) along with a pair of quartet at 7.12 (2H, dd, 7.1Hz, 7.7 Hz) and 7.05 (2H, dd, 7.1Hz, 7.8 Hz), revealed the existence of an ortho-disubistuted aromatic system. Two more singlets at 3.8 (6H) and 8.2 (2H) indicated the presence of a methoxy group and an olefinic proton attached to carboxylic group, respectively. The ^13^C NMR spectrum showed the typical signal pattern for an indole 2, 3-subistuted derivative (Table 2) and signals at 141.1, 120.5, 125.1, 138.4 and 128.2 emphasized on this finding. Moreover two signals at 51.7 and 166.7 revealed the presence of methyl ester group in the structure. Complete structure assignments were done by COSY, HMQC and HMBC experiments. HMBC correlations were observed between olefinic proton (H-10) and carbonyl resonance at 166.7. In the same way, HMBC correlation between protons of methoxy (resonating at 3.8) and carbonyl group was shown. Furthermore, HMBC correlation between H-10 and C resonance at 141.1 revealed the presence of indole- 3- subistuted moiety in the structure. Finally, relying on this evidence and by comparison of spectral data with previously published one for similar samples (33-34), the compound was identified as caulerpin )Figure 1(.

**Table 1:** Brine shrimp toxicity, antioxidant activity and cytotoxicity of the caulerpin and *C. peltata* extracts.

**Caulerpa peltata** **extract**	**BSLA** b ** LC**_50 _ **(mg/mL)**	**MTT growth inhibition (%); at 10µg/mL**	**DPPHRC**_50_ ** (mg/mL)**
Hexane	0.501	26.5	1.56
DCM^a^	2.512	33.0	1.01
Methanol	0.112	5.2	3.47
Caulerpin	**n.d.** c	12.2	**n.d.**
control^d^	0.003	0	2.9 x 10^-5^

a : Dichloromethane,

b : Brine shrimp lethality assay,

c : not determined,

d : BSLA: podophyllotoxin; MTT: kahalalide; DPPH: quercetin

**Table 2:** ^1^H and ^13^C NMR data of Caulerpin in CH_3_OH-d_4_

**Position **	**δH (J in Hz)**	**δC**
1, 1’	a	-
2, 2’	-	141.1
3, 3’	-	120.5
4, 4’	7.4 (2H, d, 8Hz )	125.1
5, 5’	7.12 (2H, dd, 7.1Hz, 7.7 Hz)	119.7
6, 6’	7.05 (2H, dd, 7.1Hz,7.8 Hz )	123.2
7, 7’	7.3 (2H, d, 8Hz )	112.3
8, 8’	-	138.4
9, 9’	-	128.2
10,10’	8.2 ( 2H, s)	130.1
11,11’	-	133.5
12, 12’ CO	-	166.7
OCH_3_	3.8 (6H, s)	51.7

Assignments were confirmed by COSY, HMQC and HMBC.

a not visible; due to the proton exchange.

*Brine Shrimp Lethality Assay (BSLA)*

The original method developed by Myer *et al.* (1982) was used by some modification to assess general toxicity of *C. peltata* extract. The obtained results were evaluated by Probit analysis and their LC_50 _value was calculated (Table 1). The LC_50_ value for the podophyllotoxin, a well-known cytotoxic plant lignan, calculated as 2.9x10^-3^ mg/mL. According to our finding the methanol extract exhibited the highest degree of toxicity with a LD50 value of 0.112 mg/mL which comparing the LC_50_ value of podophyllotoxin is not significant. The hexane extract exerted toxicity toward nauplii, but obviously not at all concentration and the DCM extract was nearly non-toxic. 

*DPPH assay*

The results of DPPH assay are summarized in Table 1. In the DPPH assay the DCM, hexane and methanol extracts showed more free radical scavenging (antioxidant) activities, respectively. Surprisingly, the methanol extract showed lowest antioxidant effect (RC_50_ =3.74 mg/mL) comparing the activity quercetin (RC50 = 2.9x10^-5^).

*MTT assay*

The antiproliferative activity of *C. peltata *extracts and c*aulerpin* was determined by MTT method (Table 1). The collected results showed that the DCM extract was more active than the hexane and methanol extracts. Despite these finding, the alga extracts could not show any significant antiproliferative effects in comparison with positive control, Kahalalide.

*Antibacterial activity*

None of the extracts and caulerpin exhibited any inhibitory activities against the applied bacteria. 

## Discussion

Present work evaluated the general toxicity, antioxidant potential, antiproliferative activity and antibacterial effects of *C. peltata* – a green alga and caulerpin. Native communities in the south-east of Iran consume this alga as medicine and food additive, yet no information regarding to its safety has been published. 

Preliminary experiments indicated that methanol extract of *Caulerpa peltata* was active against brine shrimp nauplii at nearly all applied concentrations. Bioassay guided fractionation and isolation of *C. peltata *methanol extract allowed to isolate an orange red pigment, caulerpin. Caulerpin has previously reported from *C. peltata *(20, 25) and other species of *Caulerpa* genus (16, 25, 35-37). The compound has also isolated from some other green and red algae(20). The BSLA is not specific to any pharmacological activity but can give a wide overview of activities present. Anticancer agents have been shown to be effective in the BSLA; however it is important to note that not all activity present in this assay could be anti-cancerous. Obviously the BSLA is simply used to screen substances that are toxic to zoological systems. It is note worthy that, the methanol extract of *C. peltata* in spite of exhibiting a moderate toxicity against the brine shrimp nauplii, it was nearly inactive on L5178Y mouse lymphoma cells. Moreover, caulerpin – the compound responsible for being toxic to the brine shrimp, did not show remarkable anticancer effects on L5178Y mouse lymphoma cells.

In the same manner, this result agrees with previous work that indicated the cytotoxic effect of the extract of *C. racemosa* on human melanoma amelanotic cell line C32, while isolated caulerpin did not show any cytotoxicity (33). 

On the other hand, Vidal *et al.(*1984) showed that caulerpin and caulerpicin are not responsible for the toxic effects observed after ingestion of *C. scalpelliformis *(21). Our study showed that the *C. peltata* extracts have no antibacterial effects against the gram positive and negative bacteria. Recently Brazilian scientists have found that caulerpin possess a anti HSV-1 effect (22). 

It is believed that the important part of antioxidant activity of natural products, specially phenolics, is resulting from their redox properties and to some extent, their metal chelating potential (28). Obviously caulerpin has not free phenolic group in its structure and hence could not show a dominant antioxidant activity. The RC_50_ values of the hexane, DCM and methanol extracts were found to be 1.56, 1.01 and 3.47 mg/mL, respectively which are prominently lower than the RC_50_ value of the quercetin (2.9x10^-5^ mg/mL).This *result* suggests that *C. peltata* can be used as a kind of natural food and fiber without any toxic and harmful effect to human. To the best of our knowledge, this is the first report on phytochemistry and bioactivity of *C. peltata* which collected from Oman Sea.

## References

[1] Burja AM, Banaigs B, Abou-Mansour E, Grant Burgess J, Wright PC (2001). Marine cyanobacteria—a prolific source of natural products. Tetrahedron.

[2] El Sayed KA, Bartyzel P, Shen X, Perry TL, Zjawiony JK, Hamann MT (2000). Marine natural products as antituberculosis agents. Tetrahedron.

[3] Mayer AM, Gustafson KR (2004). Marine pharmacology in 2001-2: antitumour and cytotoxic compounds. Eur. J. Cancer.

[4] Mayer AM, Hamann MT (2005). Marine pharmacology in 2001–2002: Marine compounds with anthelmintic, antibacterial, anticoagulant, antidiabetic, antifungal, anti-inflammatory, antimalarial, antiplatelet, antiprotozoal, antituberculosis, and antiviral activities; affecting the cardiovascular, immune and nervous systems and other miscellaneous mechanisms of action. Comp. Biochem. Physiol. C Toxicol. Pharmacol..

[5] Mayer AM, Gustafson KR (2006). Marine pharmacology in 2003-2004: anti-tumour and cytotoxic compounds. Eur. J. Cancer.

[6] Mayer AM, Rodriguez AD, Berlinck RG, Hamann MT (2007). Marine pharmacology in 2003-4: marine compounds with anthelmintic antibacterial, anticoagulant, antifungal, anti-inflammatory, antimalarial, antiplatelet, antiprotozoal, antituberculosis, and antiviral activities; affecting the cardiovascular, immune and nervous systems, and other miscellaneous mechanisms of action. Comp. Biochem. Physiol. C Toxicol. Pharmacol..

[7] Mayer AM, Gustafson KR (2008). Marine pharmacology in 2005-2006: antitumour and cytotoxic compounds. Eur. J. Cancer.

[8] da Rocha AB, Lopes RM, Schwartsmann G (2001). Natural products in anticancer therapy. Curr. Opin. Pharmacol..

[9] Hayat S, Atta-ur R, Choudhary MI, Khan KM, Abbaskhan A (2002). Two new cinnamic acid esters from Marine brown alga Spatoglossum variabile. Chem. Pharm. Bull Tokyo.

[10] Hegazi MM, Pérez-Ruzafa A, Almela L, María-Emilia C (1998). Separation and identification of chlorophylls and carotenoids from Caulerpa prolifera, Jania rubens and Padina pavonica by reversed-phase high-performance liquid chromatography. J. Chromatogr. A.

[11] Bhaskar N, Miyashita K (2005). Lipid composition of Padina tetratomatica (Dictyotales, Pheophyta), a brown seaweed of the west coast of India. Ind. J. Fisheries.

[12] Fleurence J (1999). Seaweed proteins: biochemical, nutritional aspects and potential uses. Trends Food Sci. Technol.

[13] Mabeau S, Fleurence J (1993). Seaweed in food products: biochemical and nutritional aspects. Trends Food Sci. Technol.

[14] McHugh DJ (1987). Production and Utilization of Products from Commercial Seaweeds.

[15] Chandini SK, Ganesan P, Bhaskar N (2008). In-vitro antioxidant activities of three selected brown seaweeds of India. Food Chem.

[16] Capon RJ, Ghisalberti EL, Jefferies PR (1983). Metabolites of the green algae, Caulerpa species. Phytochem.

[17] Bhakuni D, Rawat D (2005). Bioactive Marine Natural Products.

[18] Higa T, Kuniyoshi M (2000). Toxins associated with medicinal and edible seaweeds. Toxin. Rev.

[19] Box A, Sureda A, Tauler P, Terrados J, Marba N, Pons A (2010). Seasonality of caulerpenyne content in native Caulerpa prolifera and invasive C. taxifolia and C. racemosa var. cylindracea in the western Mediterranean Sea. Bot. Mar.

[20] Guven KC, Percot A, Sezik E (2010). Alkaloids in marine algae. Mar. Drugs.

[21] JP Vidal, D Laurent, SA Kabore, E Rechencq, M Boucard, JP Girard, R Escale, JC Rossi (1984). Caulerpin, caulerpicin, caulerpa scalpelliformis: comparative acute toxicity study. Bot. Mar.

[22] Nathália Regina Porto Vieira Macedo, Michele Ribeiro, Roberto Villaça, Wilton Ferreira, Ana Maria Pinto, Valéria L Teixeira, Claudio Cirne-Santos, Izabel PPaixão, Viveca Giongo (2012). Caulerpin as a potential antiviral drug against herpes simplex virus type 1. Braz. J. Pharmacog.

[23] Vairappan CS (2004). Antibacterial activity of major secondary metabolities: found in four species of edible green macroalgae genus Caulerpa. Asian J. Microbiol. Biotechnol. Environ. Exp. Sci.

[24] Ayyad S, Badria F (1994). Caulerpine: An antitumor indole alkaloid from Caulerpa racemosa. Alex. J. Pharm. Sci.

[25] Michael F, Raub John H, Cardellina, John G Schwedea (1987). The green algal pigment caulerpin as a plant growth regulator. Phytochem.

[26] Sohrabipour J, Rabei R, Nezhadsatari T, Asadi M (2004). The marine algae of the southern coast of Iran,Persian Gulf, Lengeh area. Iran. J. Bot.

[27] Sohrabipour J, Rabei R (1999). A list of marine algae of sea shores of the Persian Gulf and Oman Sea in the Hormozgan province. Iran. J. Bot.

[28] Shoeb M, MacManus SM, Kumarasamy Y, Jaspars M, Nahar L, Thoo-Lin PK, Nazemiyeh H, Sarker SD (2006). Americanin, a bioactive dibenzylbutyrolactone lignan, from the seeds of Centaurea americana. Phytochem.

[29] Ashour M, Edrada R, Ebel R, Wray V, Watjen W, Padmakumar K, Muller WE, Lin WH, Proksch P (2006). Kahalalide derivatives from the Indian sacoglossan mollusk Elysia grandifolia. J. Nat. Prod.

[30] Nazemiyeh H, Bahadori F, Delazar A, Ay M, Topçu G, Nahar L, Majinda RRT, Sarker SD (2008). Antioxidant phenolic compounds from the leaves of Erica Arborea (Ericaceae). Nat. Prod. Res.

[31] Nazemiyeh H, Rahman MM, Gibbons S, Nahar L, Delazar A, Ghahramani MA, Talebpour AH, Sarker SD (2008). Assessment of the antibacterial activity of phenylethanoid glycosides from Phlomis lanceolata against multiple-drug-resistant strains of Staphylococcus aureus. J. Nat. Med.

[32] Sarker SD, Nahar L, Kumarasamy Y (2007). Microtitre plate-based antibacterial assay incorporating resazurin as an indicator of cell growth, and its application in the in-vitro antibacterial screening of phytochemicals. Methods.

[33] Rocha FD, Soares AR, Houghton PJ, Pereira RC, Kaplan MA, Teixeira VL (2007). Potential cytotoxic activity of some Brazilian seaweeds on human melanoma cells. Phytother. Res.

[34] Maiti B, Thomson R, Mahendran M (1978). The structure of caulerpin, a pigment from Caulerpa algae. J. Chem. Res. Synop.

[35] Vest SE, Dawes CJ, Romeo JT (1983). Distribution of caulerpin and caulerpicin in eight species of the green alga Caulerpa (Caulerpales). Bot. Mar.

[36] Schroder HC, Badria FA, Ayyad SN, Batel R, Wiens M, Hassanein HM, Kurelec B, Muller WE (1998). Inhibitory effects of extracts from the marine alga Caulerpa taxifolia and of toxin from Caulerpa racemosa on multixenobiotic resistance in the marine sponge Geodia cydonium. Environ. Toxicol. Pharmacol.

[37] Mao SC, Guo YW, Shen X (2006). Two novel aromatic valerenane-type sesquiterpenes from the Chinese green alga Caulerpa taxifolia. Bioorg. Med. Chem. Lett.

